# Book Review: Support in Education

**DOI:** 10.3389/fpsyg.2021.696859

**Published:** 2021-08-02

**Authors:** Yonghe Ti, Jiangfeng Yang

**Affiliations:** ^1^Institute of Education, Tsinghua University, Beijing, China; ^2^College of Education, Zhejiang University, Hangzhou, China

**Keywords:** educational support, assistance, learning autonomy, support tools, higher education, inclusive education

Every student is inseparable from support. Effective support is essential for students to engage in learning positively (Kim et al., 2018; Lei et al., 2018). This book introduces several facets of efforts to enhance a support environment mainly in the French educational system and transmits the notion that education support is not there to control the learning process but to enable the autonomy of the learners to flourish.

## Evaluation of The Book's Content

The 10 chapters of this book are divided into four parts.

Part I, including the first two chapters, discusses theoretical approaches to education support. Chapter 1 outlines when students request assistance and the assistance practices used by teachers or institutions in the French education system, which emphasizes the necessity of joint efforts to construct self-regulated assistance in education. Chapter 2 discusses the two unique identities of teachers when giving assistance and their underlying logic of evaluation and accompanying aims to develop while examining guider aims (p. 42). Furthermore, it proposes how to balance these two conflicting identities under the pressure of standardized evaluation in education, which requires further research in the future (p. 46).

Part II, including the next three chapters, shows solicitude for the specific needs of particular students. Chapter 3 reports the condition of using information and communication technologies for students with visual impairment and teachers in mathematics and science classes. It highlights the importance of developing usable digital resources for students with visual impairment to support their learning autonomy. Chapter 4 focuses on the interactive process of help-seeking between students with disabilities and their teachers in the classroom. It finds that students with disabilities mainly request instrumental help and confirmation-asking (p. 94) and their construction of knowledge differs from the perception of their teachers. Therefore, it is necessary to promote the professional skills of teachers to support students with special needs in inclusive education. Chapter 5 illustrates the influence of help-seeking environments on students' motivation and help-seeking behavior in learning arts. It suggests that the digital anonymity of seeking help can promote the need for autonomy and enhance help-seeking behavior.

Part III, including Chapters 6–8, introduces the transformative tools for instruction to support autonomous learning, respectively. Chapter 6 primarily examines the dynamics of teacher's postures, which refers to how teachers position themselves as an actor in professional development (p. 125), in the distance learning environment based on the framework of six postures. Chapter 7 examines the effect of course support application to support teachers' autonomy in professional training and points out that leading teachers to enjoy their learning autonomy can “transform their pedagogical practice to serve student learning” (p. 167). Chapter 8 examines the effect of the mind map for students in integrating the relationship among conceptions and variables. It demonstrates that the mind map is an effective tool to support self-regulated learning in higher education.

Part IV, including the last two chapters, transcends the ordinary classroom and investigates the innovative forms of support in a pedagogical context. Chapter 9 examines the effect of embedded librarianship in the flipped class, indicating that it can broaden support resources and enhance student reflection (p. 210). Chapter 10 presents various types of help in a learning interactive space and finds that “help is a dynamic, fragile, individual process” (p. 227). Therefore, a deep analysis of learner's needs is the start for teachers to support the development of self-regulated learning.

## Discussion

This book illustrates the importance of improving the inner autonomy of learners, which is the fundamental goal of external support. The book avoids dogmatically interpreting the necessity of applying related theories such as self-determination or student-centered learning into educational practice. All the methods of support that are illustrated are rooted in the actual needs of different students and their teachers in the French education system. These practice-driven support approaches are inspected using empirical research and are even heuristic for teachers and researchers in other countries.

In particular, this book focuses on the specific needs of students with disabilities in French inclusive education, which is one of the reasons why this book is worth reading. It proves that support is not only an equal interactive process between teachers and students but also a reflection field used to promote equal educational opportunities for every student. This arrangement may prompt readers to examine assistance in education from the perspective of special students, which may lead them to be more aware of how unreasonable the instructions and educational equipment that ordinary students are accustomed to are and could motivate them to design a more suitable environment for inclusive education.

Although this book is heuristic, there are several shortcomings. For example, although Part III introduces the empirical research and principles of several specific tools to support learning in detail, these contents are not enough to guide teachers to use these tools in practice. That is to say, how these support tools coordinate with multiple specific teaching contents needs further elaboration. Both Parts III and IV substantially focus on support practice in higher education. Readers who would like to learn more about support practice in primary and secondary schools may only find a small amount of information. Last but not the least, this book mainly discusses support in formal education. Nevertheless, the development of a student is influenced by various hierarchical factors from the ecological perspectives and family plays an important role (Bronfenbrenner, 1986). Therefore, a second edition could add more chapters that interpret support from family or community to satisfy more potential readers.

## Author Contributions

YT wrote the manuscript and JY revised it. Both authors contributed to the article and approved the submitted version.

## Conflict of Interest

The authors declare that the research was conducted in the absence of any commercial or financial relationships that could be construed as a potential conflict of interest.

## Publisher's Note

All claims expressed in this article are solely those of the authors and do not necessarily represent those of their affiliated organizations, or those of the publisher, the editors and the reviewers. Any product that may be evaluated in this article, or claim that may be made by its manufacturer, is not guaranteed or endorsed by the publisher.
